# Focus on collagen: *in vitro *systems to study fibrogenesis and antifibrosis ^_^ state of the art

**DOI:** 10.1186/1755-1536-2-7

**Published:** 2009-12-15

**Authors:** Clarice ZC Chen, Michael Raghunath

**Affiliations:** 1 Division of Bioengineering, Faculty of Engineering, National University of Singapore, DSO Building (Kent Ridge), Medical Drive, Singapore; 2 NUS Graduate School for Integrative Sciences and Engineering, National University of Singapore, Singapore; 3 Department of Biochemistry, Yong Loo Lin School of Medicine, National University of Singapore, Singapore

## Abstract

Fibrosis represents a major global disease burden, yet a potent antifibrotic compound is still not in sight. Part of the explanation for this situation is the difficulties that both academic laboratories and research and development departments in the pharmaceutical industry have been facing in re-enacting the fibrotic process *in vitro *for screening procedures prior to animal testing. Effective *in vitro *characterization of antifibrotic compounds has been hampered by cell culture settings that are lacking crucial cofactors or are not holistic representations of the biosynthetic and depositional pathway leading to the formation of an insoluble pericellular collagen matrix. In order to appreciate the task which *in vitro *screening of antifibrotics is up against, we will first review the fibrotic process by categorizing it into events that are upstream of collagen biosynthesis and the actual biosynthetic and depositional cascade of collagen I. We point out oversights such as the omission of vitamin C, a vital cofactor for the production of stable procollagen molecules, as well as the little known *in vitro *tardy procollagen processing by collagen C-proteinase/BMP-1, another reason for minimal collagen deposition in cell culture. We review current methods of cell culture and collagen quantitation *vis-à-vis *the high content options and requirements for normalization against cell number for meaningful data retrieval. Only when collagen has formed a fibrillar matrix that becomes cross-linked, invested with ligands, and can be remodelled and resorbed, the complete picture of fibrogenesis can be reflected *in vitro*. We show here how this can be achieved. A well thought-out *in vitro *fibrogenesis system represents the missing link between brute force chemical library screens and rational animal experimentation, thus providing both cost-effectiveness and streamlined procedures towards the development of better antifibrotic drugs.

## Fibrosis - ubiquitous problem and global burden

Repair of damaged tissues is an essential biological process which allows directed replacement of dead or damaged cells with connective tissue after injury. The repaired area is addressed as a scar. Hence, scarring represents a survival mechanism that is conserved throughout evolution and appears to be most pronounced in humans. If this wound healing process goes awry, fibrosis results, often causing an excessively large scar or the scarry transformation of organ parts or whole organs. Besides local scarring at sites of acute trauma, a variety of other causes, such as chronic infections, chronic exposure to alcohol and other toxins, autoimmune and allergic reactions, radio- and chemotherapy, can all lead to fibrosis. This pathological process, therefore, can occur in almost any organ or tissue of the body and, typically, results from situations persisting for several weeks or months in which inflammation, tissue destruction and repair occur simultaneously. In this setting, fibrosis most frequently affects the lungs, liver, skin and kidneys. There are approximately 5 million cases of idiopathic lung fibrosis globally [1], not counting rare disorders like cystic fibrosis or very common ones such as asthma. Chronic hepatitis virus B and C are a major cause of liver fibrosis/cirrhosis which currently ranks 18th of the global disease burden [2]. Scar formation after myocardial infarction can on one hand prevent the injured myocardium from dilatation and rupture but, on the other hand, it can impair cardiac function through increasing ventricular wall stiffness [3]. Atherosclerotic lesions contain fibrotic tissue which can occupy up 87% of total plaque area [4].

Peri-implantational fibrosis represents a current clinical roadblock in regenerative medicine, which is gaining attention in the tissue engineering field. Every implant is surrounded by a fibrotic tissue reaction that depends on the material, its surface and its degradation profile [5-7]. This is a consequence of chronic local inflammation and a reflection of the host's tissue attempt to destroy the implant or to cope with it. If destruction is not an option, the implants get wrapped in a fibrous shroud with sparse or no vascularization, so that it becomes effectively isolated from the surrounding tissue. This is seen in artificial ligaments [8,9], implanted biosensors [10,11], joint implants [12,13], breast implants [14,15], encapsulated tissues/cells [16,17], drug delivery systems [18] and eye implants [19,20], and regularly impairs the proper function of the implant. This has prompted the field to alter surface structures and coatings to contain this problem [5,21-27]. A potential strategy could be to develop biomaterials that will deliver an antifibrotic substance locally [15,28,29].

It becomes clear that the development of effective antifibrotics is an important unmet clinical need and with it remains the necessity for rapid *in vitro *screening tools to characterize lead antifibrotic compounds before they are tested in animal models. This review will focus on the current state of the art to emulate a fibrotic process *in vitro*, the associated challenges and pitfalls and suggestions on how to address them.

## Fibrogenesis *in vivo *- complexity and key players

In order to appreciate the task which *in vitro *screening of antifibrotics is up against, we shall dissect the fibrotic process into two categories: first, events that are upstream of collagen biosynthesis; and, secondly, the biosynthetic and depositional cascade of collagen I.

### Upstream events of fibrosis - cellular players *in vivo*

Trauma disrupts the anatomical cohesion of tissue structures, most evident by bleeding which indicates breakage of blood vessels and disruption of their endothelial lining. This immediately induces a haemostatic response encompassing platelet aggregation, blood clot formation and accumulation of provisional ECM [30]. Damaged epithelia secrete cytokines, growth factors and chemoattractants for mononuclear cells to phagocytose cellular debris at the site of injury and for fibroblasts to deposit collagen and remodel it. Thus, a scar is formed that eventually matures. The origin of these fibroblasts is currently a matter of debate. They are either differentiating homing mesenchymal stem cells [31], fibrocytes from the blood circulation [32] or are derived from epithelia via epithelial-mesenchymal transition (EMT) [33]. The fibroblasts involved in scarring have a myofibroblast phenotype characterised by α-smooth muscle actin (α-SMA) expression, increased secretion of collagen type I and III, and contractility [30]. These cells are believed to be responsible for the majority of collagen production in most organs.

### 'Soluble' factors mediating fibrosis

The cellular effectors of fibrosis are activated and phenotypically modulated by humoral players, namely chemokines, growth factors and cytokines. Most notorious is transforming growth factor β1 (TGF-β1) which supports wound healing and repair. Under pathological conditions, TGF-β1 coordinates a cross-talk between parenchymal inflammatory and collagen-expressing cells, and plays a key role in fibrosis progression. TGF-β1 is often referred to as a 'soluble' factor. We use quotation marks here because TGF-β1 is stored in its latent form bound to TGF-β1 binding proteins in the matrix [34], and can in its active form be scavenged and possibly neutralized by decorin-mediated binding into the ECM [35,36] (for review see [37]).

Along with factors such as the epithelial growth factor, basic fibroblast growth factor and interleukin-1, TGF-β1 appears to play a key role in EMT [32]. The connective tissue growth factor [38] and platelet-derived growth factor [39] have also been reported to be involved in fibrosis (for a more in-depth review on cytokines and molecular mechanisms involved in fibrogenesis, refer to [40]). Inflammation typically precedes fibrosis, although it has been demonstrated that fibrosis is not always driven by inflammation. This suggests that the mechanisms that regulate fibrogenesis are, to a certain extent, distinct from those regulating inflammation [41]. This may explain the lack of efficacy of anti-inflammatory compounds in the treatment of fibrotic disease [42]. Antifibrotic strategies at the upstream level aim to interfere with fibrotic growth factors and make use of interfering antibodies [43-46], small molecules [39,47], proteins [48,49], antisense technology [50,51] or the use of human recombinant TGF-β3. TGF-β3 is an alleged TGF-β1 antagonist that has shown some promise in phase III clinical trials in a prophylactic setting of small skin wounds [52].

### Understanding the last mile of the fibrotic pathway

Irrespective of upstream events that trigger and entertain fibrosis, the final product of cellular activity is the massive deposition of collagen which results in scar formation, organ or peri-implantational fibrosis. Therefore, we would like to turn now to the obvious target in fibrosis, namely the biosynthetic pathway of collagen itself.

**Figure 1:**
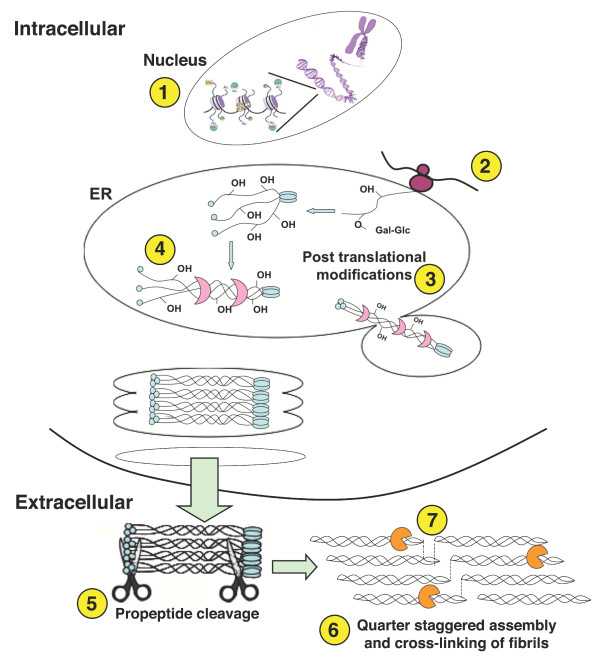
**Potential points of interference along the collagen biosynthesis pathway**. (1) Epigenetic level: HDAC inhibitors. (2) Post-transcriptional level: mRNA translation is reduced by miRNAs/siRNAs. (3) Post-translational level: prolyl-4-hydroxylase inhibitors reduce the stability of the procollagen triple helix. (4) Reduction/inhibition of the collagen chaperone hsp47 (pink crescent symbol) also reduces stability of the procollagen triple helices, resulting in intracellular retention and degradation. (5) Post-secretional level: Inhibition of procollagen proteinases (scissors symbol) prevents deposition of insoluble collagen molecules on the cell layer. (6) Collagen crosslinking: Inhibition of lysyl oxidase (LOX) hypothetically renders the collagen more susceptible to degradation. 7) An increase of MMP1 (orange Pacman symbol) results in faster collagen degradation and turnover.

The overall amount of collagen deposited by fibroblasts is a regulated balance between collagen synthesis and collagen catabolism, which is a carefully controlled process. During a pathological maturation and remodelling phase, collagen synthesized by fibroblasts exceeds the rate at which it is degraded such that the net amount of collagen continues to increase. There are several key points along the collagen biosynthesis pathway that can be targeted to effect a net reduction of collagen secretion and/or deposition (Figure 1). Transcription interference can be affected using histone deacetylase inhibitors [53,54] or substances like halofuginone [55,56]. At the post-transcriptional level, siRNA targeting growth factors and key players have been investigated [50,51,57] and, recently, we and others have suggested the use of microRNAs [58,59]. Interfering with post-translational modifications by inhibiting prolyl-4 hydroxylase renders collagen triple helices less thermostable and prevents their secretion [59,60], down-regulation of collagen chaperone hsp47 does likewise [61,62]. At the extracellular level, inhibition of procollagen C-proteinase/BMP1 prevents the removal of the C-terminal propeptide from the procollagen I molecule and, thus, the supramolecular assembly of collagen into fibrils [47,59]. Inhibition of lysyl oxidase mediation of inter-molecular cross-links between collagen triple helices can reduce collagen content of the ECM, presumably by rendering collagen aggregates more susceptible to proteolytic remodelling [63]. Similarly, the administration of hepatocyte growth factor [64] and matrix metalloproteinase 1 (MMP1) [48,49] increases collagen turnover in the ECM. It becomes clear that a meaningful *in vitro *system for the testing and characterization of antifibrotics should be able to emulate the above described complete collagen matrix formation cascade, encompassing its biosynthesis and all post-translational (intra- and extra-cellular) modifications that give rise to a stable supramolecular assembly, and also ideally allow the study of remodelling/fibrolysis. For the sake of convenience and efficiency for screening purposes, quantitation of collagen and other proteins of interest should preferentially be in and from one well.

## Fibrogenesis *in vitro *- constraints and options

The task of emulating collagen matrix formation *in vitro *has been surprisingly difficult, partly due to the omission of important cofactors and partially because of intrinsic properties of contemporary cell culture conditions that still are largely unknown. We will discuss these problems and their solutions.

### Biosynthetic issues - getting collagen made and deposited in vitro

(1) The first challenge for *in vitro *fibrogenesis is the sufficient production of collagen and its subsequent incorporation into a pericellular matrix. The omission of ascorbic acid in cell culture [38,65,66] results in minimal production and deposition of collagen on the cell layer. Ascorbate is a crucial cosubstrate of the enzymes responsible for the post-translational hydroxylation of prolyl and lysyl residues necessary for rendering the collagen triple helix thermostable, as well as for the extracellular cross-linking of collagen fibers, respectively [67,68]. In other words, scurvy can exist in cell culture. However, like its counterpart *in vivo*, it can be easily treated *in vitro *by the administration of ascorbate. On the other hand, L-ascorbic acid has a short half-life in culture and completely oxidizes after 3 days, so use of the stable form of ascorbate, such as a magnesium salt of L-ascorbic acid 2-phosphate hexahydrate, is highly recommended [67].

About 23% of the collagen molecule is composed of proline and hydroxyproline. As a non-essential amino acid, proline is synthesized from arginine/ornithine via the urea cycle and glutamate (directly or indirectly from glutamine via glutaminase) through the citric acid cycle. Clinical observations in burn patients suggest a drain of arginine, ornithine and glutamate [69], while wound fluid proline levels are at least 50% higher than plasma levels, suggesting active import of proline into the wound [70]. Providing additional proline or glutamine in the diet to enhance collagen biosynthesis, however, does not result in increased collagen accumulation. In contrast, arginine, and ornithine supplementation are most effective in increasing collagen deposition [71]. As cell culture media contains L-arginine and are usually supplemented with L-glutamine, direct proline supplementation of cell cultures in static and bioreactor conditions is not necessary for increasing collagens synthesis, as also shown recently [72]. However, systematic studies of proline-precursor supplementation *in vitro *under conditions of increased synthesis and deposition have yet to be conducted.

(2) Even under ascorbate supplementation, fibroblasts deposit only minimal amounts of secreted collagen I into their matrices. The reason for this lies in the tardy procollagen C-proteinase/BMP1 activity under current aqueous culture conditions. This results in an accumulation of unprocessed procollagen in the cell culture medium [73] where it does not belong and is discarded with every medium change. Analytical methods have, therefore, mostly focused on the convenient measurement of procollagen secreted into culture medium. This may be sufficient to assess compounds that primarily influence biosynthesis and/or secretion, but it would not allow assessment of any later step in collagen matrix formation.

**Figure 2:**
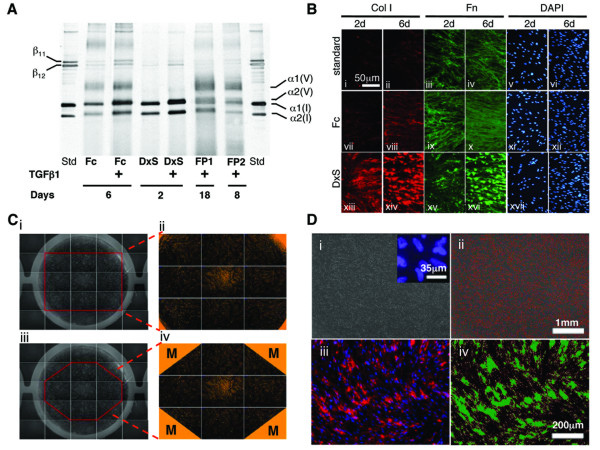
**The Scar-in-the-Jar system combines enhanced collagen deposition with optical analysis for *in situ *quantitation**. (A) Cell layers were pepsin digested, resolved by sodium dodecyl sulphate - polyacrylamide and silver stained. In comparison with fibroplasia models (FP1: Ref [61], FP2: Ref [45]), macromolecular crowding increased matrix formation including stronger lysyl oxidase-mediated cross-linking in both deposition modes (rapid: dextran sulphate [DxS]; accelerated: Ficoll cocktail [Fc]), within a shorter time frame. Note: the presence of collagen V in FP and the accelerated deposition mode and its absence in the rapid deposition mode. Collagen V is usually absent from fibrotic tissue; hence, the extracellular matrix obtained in the rapid deposition mode will probably be more similar to a fibrotic matrix. (B) Cell layers were immunostained for collagen I and fibronectin. Cell nuclei were stained with 4', 6-diamidino-2-phenylindoldilactate (DAPI). The rapid deposition mode (negatively charged, DxS) produces granular collagen I and fibronectin within 2 days, and the accelerated mode (neutral, Fc) produces collagen I with a reticular deposition pattern within 6 days. Therefore, the amount, velocity and morphology of deposited collagen can be manipulated depending on the macromolecules used. (C) Optical analysis of deposited collagen I using a 2× objective, eliminated corner auto-fluorescence in the four corner fields with triangular masks to conceal these regions during quantitation. (D) Cytometry and quantitation of the area of deposited collagen I in a 24-well multiplate format enabled identification of antifibrotic substances that perturb the collagen biosynthesis pathway resulting in a net reduction of deposited collagen I. (i) DAPI-stained nuclei at 20× total magnification in monochrome pseudocolour, 600× magnification (inset). (ii) Red scored nuclei by Count Nuclei module for cytometry. (iii) Immunostained deposited collagen I. (iv) Regions with fluorescent pixel intensity above a selected value based on controls are demarcated by the software in green for quantitation of deposited collagen I area at 100× magnification. This figure is reproduced with permission (Ref [59]).

To improve collagen deposition in cell culture, a fibroplasia model was developed using hyperconfluent human dermal fibroblasts exposed to TGF-β1 for up to 1 month to allow the formation of a fibroplastic tissue [63]. In an abbreviated form (8 days TGF-β1 treatment) this model is in use at Pfizer Global R&D (Sandwich, Kent). This model moves closer to emulating the entire collagen biosynthesis pathway as it allows the assessment of procollagen C-proteinase inhibitors, which are predicted to interfere with collagen deposition [47]. Destructive analysis of deposited collagen by high-performance liquid chromatography of 4-hydroxyproline is performed on the insoluble culture fraction. Our laboratory has systematically developed technology to accelerate collagen deposition *in vitro *by introducing macromolecules into the culture medium. We have developed two deposition technologies that differ in terms of speed and morphology of the deposited collagen. The first approach employs charged macromolecules such as dextran sulphate 500 kDa (DxS) and polysodium-4-styrene sulfonate [61]. DxS leads to a granular deposition of collagen within 48-72 h, exceeding that of non-crowded cultures by 20- to 30-fold within the same time frame. The second approach using neutral macromolecules in a Ficoll cocktail (Fc) [59] increases collagen deposition 10-fold in 6 days and in a reticular deposition pattern (Figure 2B). Both approaches are based on the creation of the excluded volume effect as explained elsewhere [61,73,74]. Briefly macromolecules drive reaction partners into closer collaboration resulting in improved protein folding and protein-protein interactions. In the case of fibrogenic cell culture, the conversion of procollagen to collagen is sped up as well as the supramolecular assembly of collagen triple helices to form fibres.

### Quantitative issues - measuring collagen and normalizing the data

(3) Determination of the amount of collagen produced *in vitro *is the next challenge, and this can be done in a variety of ways ranging from simple colorimetric assays to elaborate chromatographic procedures using radioactive and non-radioactive material. What most of these procedures have in common is the need to destroy the cell layer to obtain solubilized collagen from the pericellular matrix. The oldest established colorimetric method employs chloramine-T method to measure hydroxyproline [75]. The destructive pre-solubilization requirement of collagen for this assay, lack of specificity in discriminating between collagen types, as well as a lack of internal normalization within samples, are disadvantages in a screening setting. The *Sirius dye *has been used since 1964 to identify collagen in histology specimens [76,77]. It is based on the selective binding of Sirius Red F3BA to collagen. Subsequent elution with sodium hydroxide-methanol and read-out at 540 nm can be done in cuvettes, in microtiterplates with collagen extract adsorbed to the plastic and using microplate readers [66,78,79]. Biocolor Ltd (County Antrim, Northern Ireland, UK) made the Sircol™ Collagen Assay commercially available in 2007. Precipitation of soluble collagen with the Sirius Red dye is required prior to release of the dye with an alkali. In our hands, this assay grossly overestimates collagen and procollagen secreted into cell culture medium due to interference of non-collagenous serum proteins, so we would recommend prepurification (peptic digest and ultrafiltration) to improve both sensitivity and specificity (Ricky R Lareu, Dimitrios Zeugolis and Michael Raghunath, unpublished experiments). An interesting solution appears to be the adsorption of this dye onto collagen deposited on the cell layer in culture [80]. This was recently suggested in a modified version for the testing of antifibrotic agents [66], although this screening system did not use or recommend the addition of ascorbic acid [66]. Various enzyme-linked immunoassays can be used to quantify specific collagen types such as coating collagen extracts onto multiwell plates followed by detection using antibodies, sandwich assays and competitive enzyme immunoassays. These assays are very suitable for assessing soluble collagen from culture media but would require extraction and dialysis procedures to release insoluble collagen from pericellular matrices. In this case, normalization for differences between protein amounts or cell numbers in samples is not possible. The determination of the hydroxyproline/proline ratio via HPLC is based on total hydrolysis of protein samples, separation of hydroxyproline from proline and back calculating the possible collagen content of the sample [81]. While this method allows for the analysis of any given biological material in experienced laboratories, it is not amenable to a screening setting and would not allow for normalisation or discrimination between different collagen types, the same holds true for metabolically labelled cell cultures with the attending issues of isotope handling [82]. Similar issues arise with gas chromatography/mass spectrometry requiring derivatization using trifluoroacetylation and methanol esterification of 4-hydroxyproline in collagen. Incorporation of the stable isotope of oxygen, ^18^O_2_, into collagen is also possible with this method and enables the examination of collagen synthesis *in vitro *[83]. Polyacrylamide gel electrophoresis can be very collagen specific using metabolic labelling of cell cultures with radiolabelled amino acids glycine and proline [84] and subsequent detection of radioactive bands using fluorography [85]. Metabolic labelling can be replaced with safer and very sensitive silver staining or immunoblotting [61]. The latter has the advantage of differentiating between various collagen types and comparison with an internal standard like actin as a cell mass equivalent for normalization. Collagen antibodies tend to recognize protein conformation in addition to sequence specificity, which can pose a problem for the detection of denatured collagen α-chains in sodium dodecyl sulphate-polyacrylamide gel electrophoresis (SDS-PAGE), hence native PAGE might be an alternative option [86]. All gel electrophoresis-based approaches are excellent qualitative and quantitative back-up techniques. However, their laboriousness would exclude them as screening tools.

(4) Normalization of data to account for cell number variations is another important consideration. Most of the methods discussed above lack the option of normalization and the use of parallel cultures subjected to the separate measurement of total protein or DNA content. In any case, cell density influences the amount of collagen deposited: in fact, subconfluent cultures produce the most collagen [87]. Depending on the substance screened, inhibition or stimulation of proliferation, or cytotoxicity may occur. As this certainly will impact on the collagen amount secreted/deposited, well-to-well variances not withstanding, the acquired data must account for these changes in cell number/density. Data normalization methods involve the destruction of cell layers to quantify housekeeping proteins by Western blot or DNA [81,83]. This not only increases sample-processing steps, but also cumulates error.

### Qualitative issues - looking beyond collagen I

(5) Fibrosis is not only characterized by an excess build-up of collagen I but, depending on the tissue context, other proteins might be of interest. Also, the transition of fibrogenic cells from a quiescent fibroblast stage to a fibrotic myofibroblast phenotype should be considered. Markers such as α-SMA or fibroblast activation protein-α [88] are up-regulated in stimulated fibroblasts and a survey of the production and deposition of non-collagenous extracellular matrix (ECM) proteins as an internal control for the action of antifibrotic agents would be desirable. As well as capturing the actual number of cells in a given well, options for a high content read-out covering as many of the above markers as possible in addition to collagen I would constitute an ideal antifibrotic screening system.

## A current solution: the Scar-in-a-Jar

The Scar-in-a-Jar has been developed in our laboratory in order to address the problems of *in vitro *fibrogenesis discussed above. This system: (1) solves the problem of tardy collagen deposition; (2) quantitatively measures relative changes in collagen I deposition via immunofluorescence and *in situ *optical analysis; (3) allows for high content screening within a single well including α-SMA and another ECM protein of choice; and (4) while implementing normalization for cell number by counting nuclei via 4',6-diamidino-2-phenylindoldilactate (DAPI) staining [59]. As discussed above, the addition of charged and neutral macromolecules into culture medium dramatically enhances the deposition of collagen into the pericellular matrix [61] (Figure 2B), within a shorter time, to a greater extent and degree of cross-linking than possible in both fibroplasia models by Clark *et al*. (1997) [63] and Fish *et al*. (2007) [47] (Figure 2A). In both cases, enhanced collagen deposition reduces culture time to 48 h (DxS) and 6 days (Fc), prior to optical quantitation. TGF-β1 addition is optional. For optical analysis, samples in a 24-well format are immunostained for collagen I and fibronectin, and cell nuclei are stained with DAPI. Automated image acquisition using a 2× objective is performed and the area of collagen I and fibronectin per cell is ascertained using the Metamorph^® ^Imaging System software (Figure 2C-D).

The Scar-in-a-Jar enables the analysis of proteins-of-interest and cell enumeration all within a single-well, thus minimizing processing steps, material loss and sample variation. Cell enumeration can give an indication of potential anti-proliferative effects of a compound and allows for correction of protein production to account for the variance in cell numbers due to that compound. We also demonstrated its capability to discern the reduction of deposited collagen by known and novel inhibitors targeting various points of the collagen biosynthesis pathway from the epigenetic to the extracellular level, including two C-proteinase inhibitors, FSY002, originally developed in a German pharmaceutical company [89] and PCP56 from Pfizer (NY, USA) [47]. PCP56 demonstrated efficacy in agreement with an earlier report on the abridged fibroplasia model [47] but FSY002 did not work in our system. FSY002 has an interesting history. This phosphinate inhibitor did inhibit purified C-proteinase in the test tube and an IC_50 _value could be obtained [89]. However, tests in conventional monolayer fibroblast culture proved inconclusive and we have to conclude in hindsight that this was due to tardy C-proteinase activity. Running this substance in our system with fully active C-proteinase activity revealed its ineffectiveness. At this point in time, optical analysis is unable to discern a reduction in collagen cross-links if there is no net collagen reduction on the cell layer [59]. Hence, the testing of lysyl oxidase inhibitors will be better analysed by the biochemical analysis method employing pepsin digestion of cell layers followed by SDS-PAGE and silver staining to visualize collagen cross-links (Figure 2A) [85].

The combination of short culture time, rapid collagen biosynthesis, complete deposition, together with optical analysis that circumvents the need for protein extraction, makes the Scar-in-a-Jar a convenient assay that remains in a single well from start to finish.

### Future developments

Besides the current use of fibroblasts, this system has the flexibility and potential to screen the effect of potential antifibrotic compounds on other organ specific culprits such as hepatic stellate cells. The optical accessibility of the Scar-in-a-Jar has room for the introduction of one or more additional cell types like monocytes or smooth muscle cells to augment the fibrotic context and tailor it for a dermal wound healing situation or an atherosclerotic plaque. This could be done in direct coculture or using inserts, with the fibrotic target cells adhering to the bottom of the well. Besides preliminary testing of exogenously added matrix metalloproteinases [59], the current system has not yet been fathomed for its ability to study collagen matrix metalloproteinases turnover that would be relevant for analysing effects of collagen cross-link inhibitors and inducers of matrix metalloprtoteinases like hepatocyte growth factor. We envision longer observation times in this instance or challenging cell cultures after an initial ECM build-up, with additional cell types or substances that induce remodelling. A very promising line of research using this system could be non-enzymatic glycation studies to capture a diabetic situation and testing breakers of advanced glycation end products.

## Conclusions

With the current burden of fibrosis worldwide and acquired connective tissue disorders as seen in diabetes, the development of *in vitro *test systems that mimic fibrosis and connective tissue formation is more needed than ever. The lack of adequate systems to study fibrogenesis *in vitro *has either impeded the development of antifibrotics or has forced research and development to move too early into animal models. Although *in vivo *evaluation is pivotal for preclinical development, a rapid *in vitro *quantitative screening method is mandatory for the first round determination of potential anti-fibrotic compounds, since any animal model represents the same complexity as a human and unclear *in vitro *data will unlikely be cleared in vivo. As there are several key points along the collagen biosynthesis pathway that can be interfered with, it is necessary to emulate all of these steps *in vitro *and, ideally, to get high content information out of a single well. This problem has been finally solved with the Scar-in-a-Jar. However, we look forward with great interest to seeing refinements and further developments of antifibrotics screening in the quest for the most potent and versatile antifibrotic compound.

## Abbreviations

DAPI: 4',6-diamidino-2-phenylindole; DxS: dextran sulphate; ECM: extracellular matrix; EMT: epithelial-mesenchymal transition; Fc: Ficoll cocktail; HPLC: high-performance liquid chromatography; PAGE: polyacrylamide gel electrophoresis; SDS: sodium dodecyl sulphate; SMA: smooth muscle actin; TGF: transforming growth factor.

## Competing interests

The authors declare that they have no competing interests.

## Authors' contributions

Both CZCC and MR contributed equally to the writing of this manuscript.
